# Tbet Expression in Regulatory T Cells Is Required to Initiate Th1-Mediated Colitis

**DOI:** 10.3389/fimmu.2019.02158

**Published:** 2019-09-11

**Authors:** Martina Di Giovangiulio, Angelamaria Rizzo, Eleonora Franzè, Flavio Caprioli, Federica Facciotti, Sara Onali, Agnese Favale, Carmine Stolfi, Hans-Joerg Fehling, Giovanni Monteleone, Massimo C. Fantini

**Affiliations:** ^1^Department of Systems Medicine, University of Rome “Tor Vergata”, Rome, Italy; ^2^Gastroenterology and Endoscopy Unit, IRCCS Cà Granda Fundation, Ospedale Maggiore Policlinico, Milan, Italy; ^3^Department of Experimental Oncology, IEO European Institute of Oncology IRCCS, Milan, Italy; ^4^Department of Biomedicine and Prevention, University of Rome “Tor Vergata”, Rome, Italy; ^5^Institute of Immunology, University Clinics Ulm, Ulm, Germany

**Keywords:** Treg cells, Tbet, Th1-like Tregs, inflammatory bowel disease, inflammation

## Abstract

In normal conditions gut homeostasis is maintained by the suppressive activity of regulatory T cells (Tregs), characterized by the expression of the transcription factor FoxP3. In human inflammatory bowel disease, which is believed to be the consequence of the loss of tolerance toward antigens normally contained in the gut lumen, Tregs have been found to be increased and functionally active, thus pointing against their possible role in the pathogenesis of this immune-mediated disease. Though, in inflammatory conditions, Tregs have been shown to upregulate the T helper (Th) type 1-related transcription factor Tbet and to express the pro-inflammatory cytokine IFNγ, thus suggesting that at a certain point of the inflammatory process, Tregs might contribute to inflammation rather than suppress it. Starting from the observation that Tregs isolated from the lamina propria of active but not inactive IBD patients or uninflamed controls express Tbet and IFNγ, we investigated the functional role of Th1-like Tregs in the dextran sulfate model of colitis. As observed in human IBD, Th1-like Tregs were upregulated in the inflamed lamina propria of treated mice and the expression of Tbet and IFNγ in Tregs preceded the accumulation of conventional Th1 cells. By using a Treg-specific Tbet conditional knockout, we demonstrated that Tbet expression in Tregs is required for the development of colitis. Indeed, Tbet knockout mice developed milder colitis and showed an impaired Th1 immune response. In these mice not only the Tbet deficient Tregs but also the Tbet proficient conventional T cells showed reduced IFNγ expression. However, Tbet deficiency did not affect the Tregs suppressive capacity *in vitro* and *in vivo* in the adoptive transfer model of colitis. In conclusion here we show that Tbet expression by Tregs sustains the early phase of the Th1-mediated inflammatory response in the gut.

## Introduction

In homeostatic conditions, the gastrointestinal tract is patrolled by immune cells which are in charge to fight against pathogens. At the same time, the intestinal mucosal immune system tolerates harmless antigens contained in the gut lumen. The unbalance between these pro-inflammatory and tolerogenic activities is believed to cause inflammatory bowel disease (IBD), whose main forms are Crohn's disease (CD) and ulcerative colitis (UC). In IBD, antigens normally contained in the gut lumen derived from the gut microbiota or introduced with diet, cause an excessive activation of the immune system leading to chronic inflammation and irreversible tissue damage (1).

Regulatory T cells (Tregs) are a class of T cells which negatively control the pro-inflammatory activity of adaptive and innate immune cells and they play a key role in the maintenance of the gut immune homeostasis (2). In the absence of Tregs, as observed in case of loss-of-function mutations of the Tregs lineage committing transcription factor *foxp3* (i.e., IPEX syndrome in humans, *scurfy* mice) (3, 4), or in case of functional mutations of genes encoding for molecules involved in the Tregs suppressive activity such as CTLA4 and IL10, an uncontrolled activation of the immune system in the gut is invariantly observed (5–7).

Despite the pivotal role of Tregs in the maintenance of gut homeostasis, the number of Tregs is not reduced in the lamina propria of IBD patients and they result even increased in the inflamed areas (8, 9). Moreover, Tregs isolated from IBD patients were shown to be as suppressive as Tregs isolated from non-IBD controls (10). Therefore, the role of Tregs in the pathogenesis of IBD remains elusive.

Although considered finally differentiated cells, Tregs have been recently shown to have a certain functional plasticity. For instance, Tregs can acquire the expression of the master transcription factors that define the T helper cell subsets they are suppressing (11, 12). In this context, the expression of Tbet by Tregs, as observed in Th1 immune responses induced by *Mycobacterium* sp. infection, has been shown to induce the expression of the chemokine receptor CXCR3 and to promote the Tregs homing at the site where Th1 cells need to be kept in check (13).

Although Tbet expressing Tregs do not normally secrete pro-inflammatory cytokines and maintain their suppressive capacity, in certain conditions they can acquire a Th1-like phenotype characterized by IFNγ secretion and pro-inflammatory functions (14, 15). Moreover, several reports indicate that IFNγ -expressing Th1-like Tregs are involved in the pathogenesis of different inflammatory diseases (16–18). Although IFNγ expressing Th1-like Tregs have been described in models of intestinal inflammation (19, 20), the presence of these cells in human IBD and their functional role in intestinal inflammation remain unclear.

Here, we provide evidence that IFNγ-expressing cells accumulate in the inflamed tissue of both CD and UC patients. We also demonstrate that mice developing chemically-induced colitis are characterized by an increased number of IFNγ-expressing Th1-like Tregs in the intestinal lamina propria and that their upregulation precedes the accumulation of conventional Th1 cells. Finally, by generating Treg-specific Tbet conditional knockout mice, we demonstrate that pro-inflammatory Th1-like Tregs are required for the development of intestinal inflammation.

## Materials and Methods

### Patients

Intestinal biopsies of IBD patients (Ileal CD *n* = 8; colonic CD *n* = 5; active UC *n* = 7; inactive UC *n* = 5), and patients undergoing intestinal surgical resection for pathologies unrelated to IBD, including intestinal tumors (ileal controls *n* = 7; colonic controls *n* = 5) were obtained from the Policlinico Tor Vergata, Rome, Italy and IRCCS Ospedale Maggiore Policlinico di Milano, Milan, Italy. The clinical characteristics and concomitant therapies of IBD patients are summarized in the Supplementary Table 1. Disease extent of UC and localization and behavior of CD were described according to the Montreal classification for IBD. Local Ethics Committees (Tor Vergata University Hospital, Rome. Protocol number:154/12).

### Mice

All mice used were on C57BL6 genetic background and were housed and bred under specific pathogen-free conditions in a facility located in Castel Romano, Rome. To generate the FoxP3 reporter mice, FoxP3eGFP-Cre knock-in mice (kindly provided by A. Rudensky, MSKCC, NY, USA) which bear the coding sequence for the green fluorescent protein/iCre-recombinase fusion protein knocked-in the FoxP3 locus were crossed with the Rosa26-tdRFP mice (kindly provided by Hans Joerg Fehling, University of Ulm, Germany). tdRFP mice bear a mutation consisting in a reporter allele for Cre activity that expresses a non-toxic tandem-dimer red fluorescent protein (tdRFP) following Cre-mediated deletion of a floxed neo/stop cassette. Treg-specific Tbx21 conditional knockout mice were generated crossing FoxP3 reporter mice with mice bearing the floxed Tbx21 allele (Tbx21^fl/fl^ JAX, Bar Harbor, ME, USA). In these mice, Cre expression, under control of the FoxP3 promoter, cause the deletion of Tbx21 floxed exons leading to gene inactivation selectively in Tregs. Both FoxP3 reporter and Treg specific Tbet conditional knockout mouse strains were vital and born in the expected mendelian ratios with sign of spontaneous disease up to 12 months. IFNγ knockout mice were purchased from Jackson Laboratories (JAX, Bar Harbor, ME, USA). All animal experiments were performed in accordance with the local institutional guidelines. Male mice (6–8 weeks old) were used for all the experiments.

Authorization No: 324/2006-PR issued by the Ministry of Health on 29/03/2016 and in compliance with European rules (2010/63/UE).

### Experimental Colitis

Chemically-induced colitis was induced in FoxP3 reporter, conditional Tbx21 knockout and IFNγ knockout mice. Mice were treated with 2% dextran sulfate sodium (DSS, TdB Consultancy AB, Uppsala, Sweden) in drinking water for 7 days followed by normal drinking water until day 10. Body weight was monitored daily.

Adoptive transfer model of colitis was performed by injecting i.p. 6–8 weeks old RAG1-deficient mice with 4 × 10^5^ FACS sorted CD4+CD45RB^high^ cells from splenocytes of wild type C57BL6 mice alone or together with the same number of Tregs isolated from the spleen of FoxP3^Cre^Tbx21^wt/wt^ or FoxP3CreTbx21^fl/fl^ mice. Body weight was monitored every other day.

### Endoscopic Procedures

The development of DSS-induced colitis in mice was assessed by micro-endoscopy as previously described (21).

### Histologic Analysis of Colon Cross Sections

Colonic cross sections were stained with H&E, and the severity of inflammation was evaluated as previously described (22).

### Isolation of Leukocyte Subpopulations and Flow Cytometry Analysis

Human lamina propria mononuclear cells (LPMC) were isolated according to standard protocols. Briefly, the dissected intestinal mucosa was freed of mucus and epithelial cells in sequential steps with DTT (0.1 mmol/L) and EDTA (1 mmol/L) (both from Sigma, Milan, Italy) and then digested with collagenase D (400 U/ml) (Worthington Biochemical Corporation, Lakewood, NJ) for 5 h at 37°C. LPMC were then separated with a Percoll gradient and cultured in complete RPMI 1640 medium containing 5% human serum (Sigma, Milan, Italy) and 100 U/ml IL-2 (Proleukin, Novartis, Switzerland). Cells suspension was stained with anti-CD4 clone RPA-T4 (Biolegend, San Diego, California), anti-Foxp3 clone PCH101 (eBioscience, Thermo Fisher scientific, Italy), anti-Tbet clone 04-46 (BD Pharmigen, Milan, Italy) and anti IFNγ clone 4s.b3 (Biolegend, San Diego, California) after 5 h stimulation with 40 ng/ml phorbol 12-myristate 13-acetate (PMA) and 1 μg/ml ionomycin (Sigma Aldrich, Milan, Italy), in the presence of 2 nmol/l monensin (eBioscience, Thermo Fisher, Italy) according to standard protocols.

Mouse LPMC were obtained according to Lamina Propria Dissociation Kit mouse protocol (Miltenyi Biotech, Bergisch-Gladbach, Germany). Colonic Lamina Propria Mononuclear Cells (LPMC) were stained with LIVE/DEAD® staining (Life Technology, Milan, Italy) and with surface fluorochrome-conjugated antibodies against CD3 clone 500A2, CD4 clone GK1.5 (BD Pharmingen, Milan, Italy). Permeabilization and intracellular staining with conjugated anti-Tbet clone Q313778, anti-IFNγ clone XMG1.2 or anti-IL10 clone JES5-16E3 (BD Pharmingen, Milan, Italy), were performed after 5 h stimulation with PMA/ionomycicn. Cells were acquired with a FACS VERSE® (BD Bioscience, San Jose, CA) gating on living cells and the data were analyzed with FlowJo software (BD).

### *In vitro* Induction of Th1-Like Tregs

In some experiments CD4+ T cells were magnetically sorted from splenocytes by using the CD4+ T Cell Isolation kit mouse (Miltenyi Biotec, Bergisch Gladbach, Germany) according to the manufacture's protocol. At least 97% purity was verified by flow cytometry before each experiment. To induce Tbet and IFNγ expression, CD4+ T cells were polyclonally activated in RPMI 1640 10% FBS (Lonza, Basel, Switzerland) with plate bound anti-CD3 (clone 145-2C11, eBioscience, Thermo Fisher scientific, Italy) and 1 mg/ml anti CD28 (clone 37.31, eBioscience, Thermo Fisher scientific, Italy). In some experiment CD4+ T cells were cultured in the presence of 10 mg/ml anti mouse IFNγ neutralizing antibody (clone XMG1.2, eBioscience, Thermo Fisher scientific, Italy) or the isotype control IgG2a. FACS sorted FoxP3+ Tregs were cultured in RPMI1640 10% FBS for 24 h in the presence of plate bound anti-CD3, 10 μg/ml anti-CD28, 20 ng/ml mouse recombinant (mr)IL2 (R&D Systems, Abingdon, UK) and 100 ng/ml mrIFNγ or 15 ng/ml mrIL12 or 10 ng/ml mrIL23 or 20 ng/ml mrIL6 or 50 ng/ml mrIL21 (R&D Systems, Abingdon, UK).

### Cell Proliferation Assay

105 FACS-sorted CD4+CD45RBhigh responder (R) cells were labeled with CellTrace®-violet (Thermo Fisher scientific, Italy) according to manufacturer's instruction and co-cultured with sorted CD4+FoxP3+ Tregs suppressor (S) from splenocytes of FoxP3^Cre^Tbx21^wt/wt^ or FoxP3CreTbx21^fl/fl^ at different ratios in the presence of plate bound anti-CD3 clone 145-2C11 (BD Pharmigen, Milan, Italy) and 10^4^ sorted CD90- splenocytes used as antigen-presenting cells. CellTrace® fluorescence was evaluated after 5 days co-culture and the fraction of non-proliferating cells used as suppression index.

### RNA Extraction, Complementary DNA Preparation, and Real-Time PCR

Total RNA was isolated with the PureLink® RNA Micro Kit (Thermo Fisher scientific, Italy) for *in vitro* experiments and with PureLink® RNA Mini Kit (Thermo Fisher scientific, Italy) for tissue, according to the manufacturer's recommendations. Reverse transcription into cDNA was performed with the Superscript III Reverse Transcriptase kit (Invitrogen, Thermo Fisher scientific, Italy) according to the manufacturer's protocol and then amplified by real-time PCR using iQ SYBR Green Supermix (Bio-Rad Laboratories, Milan, Italy). PCR was performed by using the primers described in the Supplementary Table 2. RNA expression was calculated relative to the housekeeping beta-actin gene expression on the base of the ΔCt algorithm.

### Statistics

The Mann Whitney test and the unpaired student's *t*-test were used to evaluate differences between the two experimental groups after checking for normal distribution of data. Statistically significant differences between groups are indicated (*P < 0.05; **P < 0.01; ***P < 0.001). Statistical analysis was done performed using GraphPad Prism software (Graphpad Software Inc., San Diego, CA, USA).

## Results

### Th1-Like Tregs Are Up-Regulated in Active IBD

**Figure 1:**
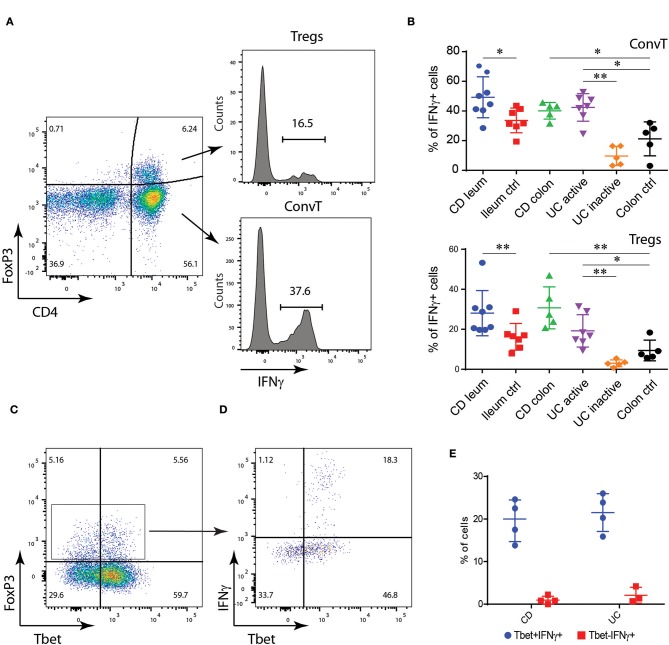
Lamina propria (LP) FoxP3+CD4+ T cells from IBD patients express IFNγ. **(A)** Flow cytometric analysis of IFNγ expression in CD4+FoxP3+ regulatory T cells (Tregs) and CD4+FoxP3- conventional T cells (ConvT) cells from LP cells of IBD patients and controls: gating strategy. **(B)** Flow cytometric analysis of IFNγ expression in Tregs (upper panel) and ConvT (lower panel) from ileal Crohn's disease (CD; *n* = 8), control ileum (*n* = 7), colonic CD (*n* = 5), active ulcerative colitis (UC; *n* = 7), inactive UC (*n* = 4), and control colon (*n* = 5). **(C,D)** Representative dot plot representing FoxP3 and Tbet expression in LP CD4+ T cells **(C)** and Tbet and IFNγ expression in LP FoxP3+CD4+ T cells **(D)** from IBD patients. **(E)** Frequency of IFNγ+ cells among Tbet+ and Tbet- Tregs in CD (*n* = 4) and UC (*n* = 3) patients. Numbers in the dot plot quadrants and histograms represent the relative frequency of cell subpopulations. Horizontal bars in **(B,D)** represent the mean value ±SD. *p < 0.05; **p < 0.01.

In a first set of experiments we aimed to assess the presence of Th1-like Tregs, characterized by the expression of Tbet and IFN-γ in the gut of IBD patients. To this end, mucosa biopsies were collected from the most inflamed intestinal areas of UC (*n* = 7) and CD (*n* = 8) patients. Biopsies from inactive UC (*n* = 5) were also collected. As controls, samples of normal ileal (*n* = 7) and colonic mucosa (*n* = 5) were taken from patients undergoing surgical resection for colonic neoplasia. As shown in Figure 1A, IFNγ was expressed in both FoxP3 negative conventional T cells (ConvT) and Foxp3+ Tregs. As expected, ConvT cells from CD patients expressed more IFNγ as compared to controls. Similarly, ConvT cells from active UC expressed more IFNγ as compared to both inactive UC and control samples (Figure 1B, upper panel). Concerning Tregs, about 30% of the FoxP3+ cells from the inflamed ileal and colonic mucosa of CD patients expressed IFNγ and their frequency was significantly higher than their relative controls. In UC, the frequency of Tregs-expressing IFNγ was about 20% and this was significantly higher than that observed in both inactive UC patients and colonic controls (Figure 1B, lower panel). Similarly to ConvT cells, a proportion of Foxp3+Tregs from inflamed IBD co-expressed the Th1-related transcription factor Tbet (Figure 1C) but IFNγ was exclusively expressed by Tbet+ Tregs in both CD and UC (Figures 1D,E). These results suggest that Th1-like Tregs, characterized by the expression of Tbet and IFNγ, might play a role during the inflammatory flares in IBD patients.

### Th1-Like Tregs Are Upregulated in the Early Phase of Inflammation in the DSS Model of Colitis

**Figure 2:**
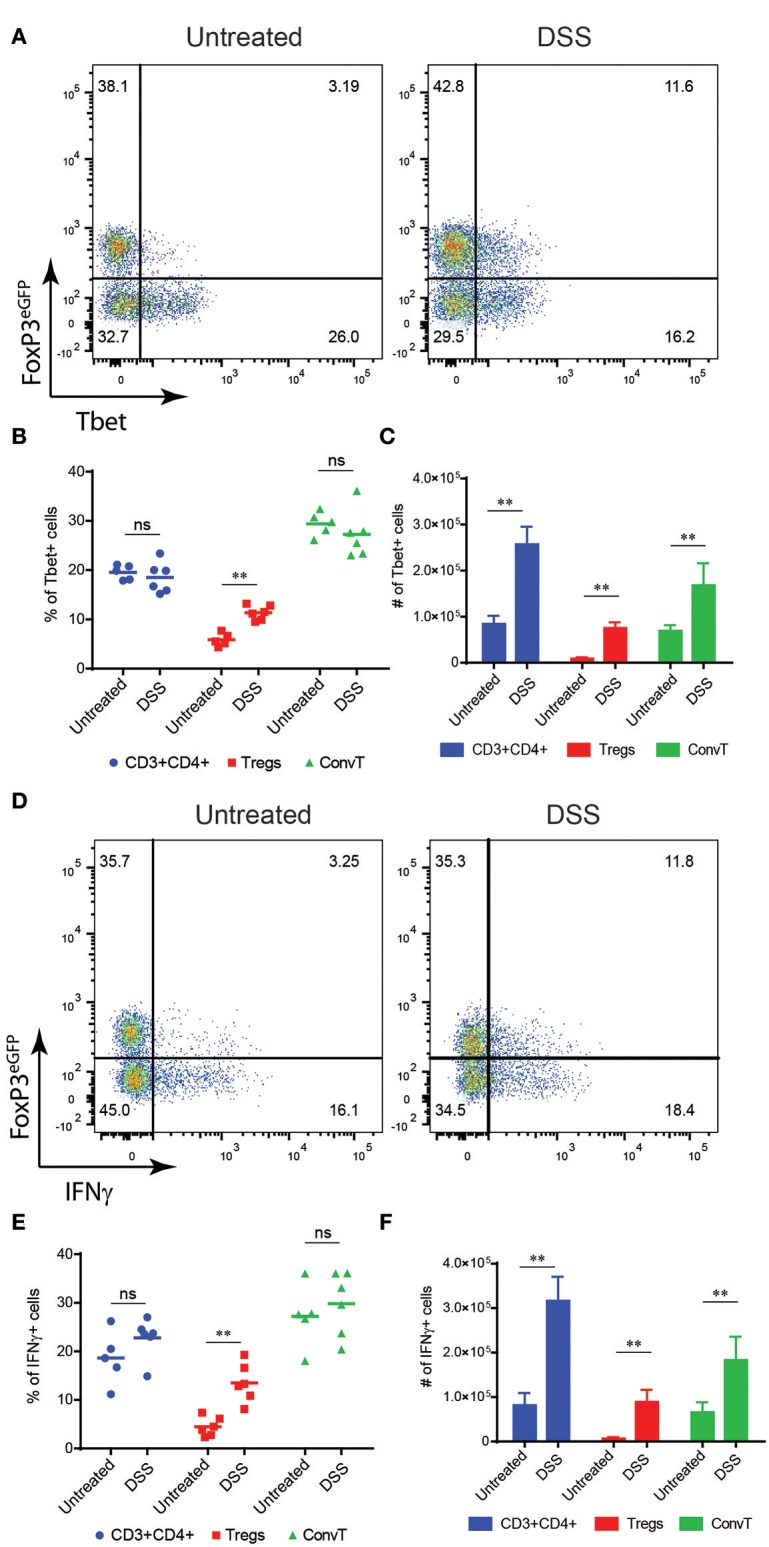
**(A)** Representative dot plots showing FoxP3^eGFP^ and Tbet expression in LP CD3+CD4+ T cells isolated from untreated and DSS-treated mice. Frequency **(B)** and absolute numbers **(C)** of Tbet+ cells among total CD4+, Treg and ConvT cells as indicated. **(D)** Representative FoxP3^eGFP^ and IFNγ expression of the same cells as in **(A)**. Frequency **(E)** and absolute **(F)** numbers of IFNγ+ cells among total CD4+, Treg, and ConvT cells as indicated. Numbers in the dot plot quadrants represent the relative frequency of cell subpopulations. Horizontal bars indicate the mean, symbols indicate each analyzed mouse. Vertical bars indicate mean ±SEM. **p < 0.01, ns, not significant.

**Figure 3:**
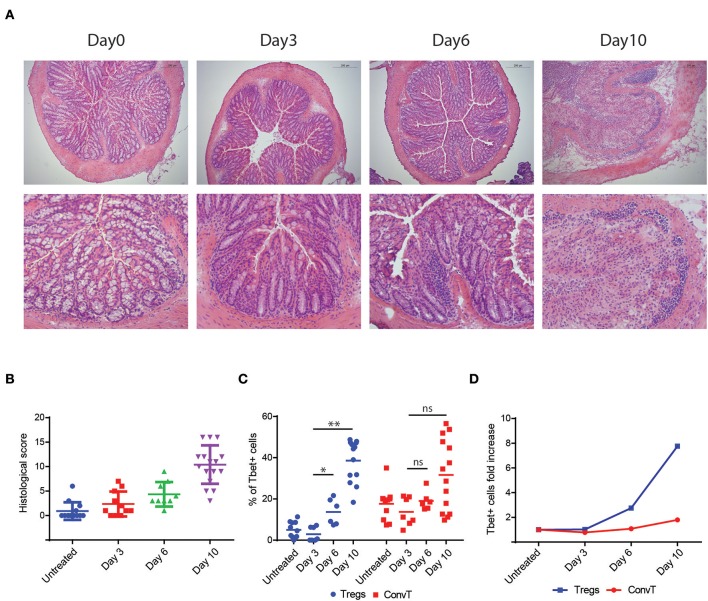
**(A)** Representative histological sections of colons from DSS-treated mice (upper panel 20x magnification, lower panel 40x magnification) at different time points as indicated. Bars in the pictures indicate the scale. **(B)** Cumulative histologic score of DSS-treated mice pooled from three independent experiments at different time points, bars indicate the mean ±SD. **(C)** Percent of Tbet+ cells among LP Tregs and ConvT cells from DSS-treated mice at different time points, symbols indicate each analyzed mouse, bars indicate the mean. **(D)** Tbet+ cells fold increase relative to baseline (day 0) in Tregs and ConvT cells. Numbers in the dot plot quadrants represent the cell relative frequency. *p < 0.05; **p < 0.01; ns, not significant.

To investigate the functional role of Th1-like Tregs in the development of intestinal inflammation, we first assessed whether Tbet-expressing Tregs were generated during intestinal inflammation induced in mice after oral administration of dextran sodium sulfate (DSS). This model is characterized by the induction of a potent Th1 immune response secondary to the damage of the intestinal epithelial barrier and increased permeability to luminal antigens. To this end, we used FoxP3^GFP−Cre^Rosa26^tdRFP^ fate mapping reporter mice. In these mice, Tregs are characterized by the double expression of eGFP and tdRFP fluorescence (Supplementary Figures 1A,B). Reporter mice were treated with DSS for 7 days and sacrificed at day 10, when the peak of inflammation occurred (Supplementary Figure 1C). At the end of the experiment, Tbet-expressing Tregs increased in the lamina propria of DSS treated mice as compared to untreated controls (Figure 2A). Interestingly, in this early phase of colitis, the frequency of Tbet positive cells among Treg but not eGFP/tdRFP double negative ConvT cells increased during inflammation (Figure 2B). However, in terms of absolute numbers, both Tbet+ ConvT and Treg cells resulted increased in treated mice as compared to controls (Figure 2C) thus suggesting that the accumulation of Tbet-expressing Tregs in the lamina propria during the initial phase of inflammation might be proportionally greater than that of ConvT cells. Accordingly, the number of Tbet+ Tregs represented one third of all the CD4+ Tbet+ cells infiltrating the lamina propria at this time point as compared to one tenth in the untreated mice. Since IFNγ expression distinguishes Tbet-expressing Tregs with Th1-specific suppressive capacity from Th1-like pro-inflammatory Tregs, we assessed IFNγ expression by lamina propria Tregs in our model. The frequency of IFNγ positive cells resulted increased among Tregs in DSS treated mice as compared to the untreated controls (Figure 2D). Similarly to Tbet expression, the frequency of IFNγ -positive cells remained stable among ConvT cells (Figure 2E) while the absolute number of both ConvT and Treg cells expressing IFNγ resulted increased in the treated mice, the Th1-like Tregs representing one third of the total IFNγ-secreting CD4+ T cells (Figure 2F). Taken together, these data indicate that in a colitis model characterized by the alteration of the epithelial barrier, Tregs acquire a Th1-like phenotype characterized by Tbet and IFNγ expression. In order to investigate how early Th1-like Tregs appeared during colitis development, we performed a time course experiment in which mice were sacrificed at day 3, 6, and 10 of the DSS protocol. At day 3 and 6, DSS treated mice were characterized by none or mild colitis as shown by histological score of colitis severity (Figures 3A,B). The frequency of Tbet-expressing Tregs but not ConvT cells increased by day 6 as compared to untreated mice when only mild signs of colitis were observed (Figures 3C,D) prompting us to hypothesize that Th1-like Tregs might represent a “ready-to-use” source of IFNγ supporting the early stage of Th1-mediated immune response.

### IFN-γ Plays a Non-redundant Role in the Induction of Th1-Like Tregs Both *in vitro* and *in vivo*

**Figure 4:**
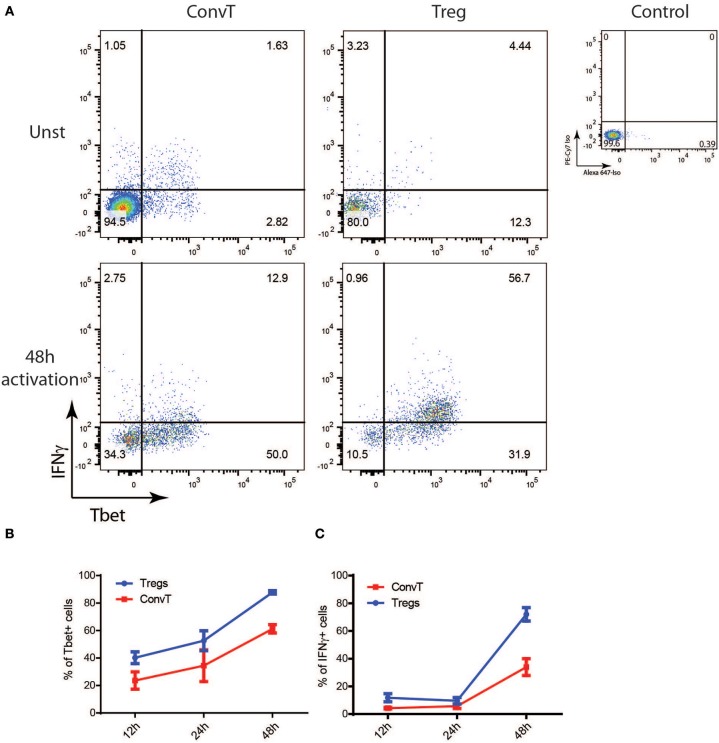
Total CD3+CD4+ T cells from the spleen of reporter mice were polyclonally activated *in vitro*. **(A)** Representative dot plots representing Tbet and IFNγ expression gating on Tregs and ConvT after 48 h of stimulation. Tbet and IFNγ isotype control stainings gating on CD4+ cells are shown (upper right plot). Percent of Tbet+ **(B)** and IFNγ+ **(C)** cells among Treg and ConvT cells at different time points. Each point represents the mean ±SD of three independent experiment performed.

**Figure 5:**
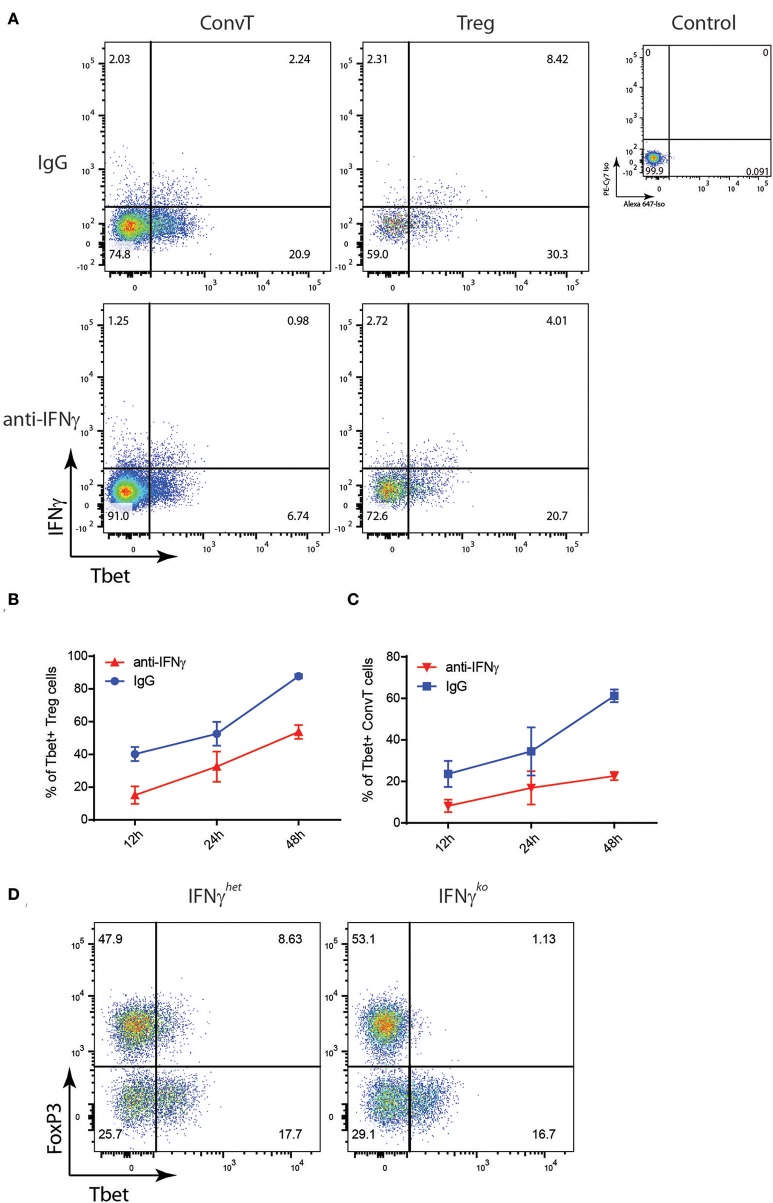
Total CD3+CD4+ T cells from the spleen of reporter mice were polyclonally activated in the presence of neutralizing anti-IFNγ antibody or control IgG. **(A)** Representative dot plots showing Tbet and IFNγ expression on gated Treg and ConvT cells after 24 h of stimulation. Tbet and IFNγ isotype control stainings gating on CD4+ cells are shown (upper right plot). **(B,C)** Percent of Tbet+ cells among Treg **(B)** and ConvT **(C)** cells in the presence of anti-IFNγ or control IgG at different time points. Each point represents the mean ±SD of three experiment performed. **(D)** Representative dot plots showing FoxP3 and Tbet expression in LP CD3+CD4+ T cells of DSS-treated IFNγ^ko^ and IFNγ^het^ mice. Numbers in the dot plot quadrants represent the relative frequency of cell subpopulations.

To test the hypothesis that Tbet induction and IFNγ expression in Tregs might be induced during T cell activation to support the development of Th1 cells, total CD4+ T cells sorted from the spleen of FoxP3^GFP−Cre^Rosa26^tdRFP^ were polyclonally activated *in vitro* for 48 h or left unstimulated, and Tbet and IFNγ expression evaluated by flow cytometry. Activated but not resting cells upregulated Tbet and IFNγ in both Treg and ConvT cells (Figure 4A). Tbet resulted upregulated as early as 12 h after stimulation in both Tregs and ConvT and their relative number progressively increased reaching 85 and 60%, respectively, at 48 h (Figure 4B). Tbet expression was higher among Tregs than ConvT cells at each time point. IFNγ was expressed by a small fraction of cells after 12 and 24 h and increased at 48 h (Figure 4C). At this time point, 65 and 25% of Tregs and ConvT cells, respectively, resulted IFNγ positive. These results indicate that in the gut, Tregs are poised to rapidly upregulate Tbet and IFNγ after activation. IFNγ has been shown to induce the expression of Tbet in FoxP3+ cells. Accordingly, sorted FoxP3+ Tregs failed to upregulate Tbet after activation in the absence of IFNγ, while the addition of IFNγ to the cell culture medium but not other cytokines (i.e., IL12, IL23, IL6, or IL23), induced Tbet expression (Supplementary Figure 2A). To assess whether IFNγ was required to induce Tbet expression in our *in vitro* system, CD4+ T cells were activated in the presence or absence of neutralizing anti-IFNγ antibodies. In the presence of anti-IFNγ, Tbet expression and IFNγ secretion were reduced in both Tregs and ConvT, being the suppressive effect prevalent in the latter (Figure 5A). Moreover, the magnitude of IFNγ suppression among ConvT cells resulted bigger than the one observed in Tregs at each of the analyzed time points (Figures 5B,C).

To confirm *in vivo* the role of IFNγ in the induction of Th1-like Tregs during colitis, IFNγ^het^ and IFNγ^Ko^ mice underwent the DSS protocol and LPMC were analyzed at day 10. As previously reported, IFNγ^Ko^ mice developed milder disease as shown by the lower weight loss as compared to the IFNγ^Het^ control mice [Supplementary Figure 2B; (23)]. At the end of the experiment, Tbet expression resulted suppressed in the lamina propria Tregs of IFNγ^Ko^ mice as compared to the heterozygous controls (Figure 5D) thus demonstrating that IFNγ plays a non-redundant role in the induction of Th1-like Tregs during intestinal inflammation.

### Th1-Like Tregs Are Required for the Development of DSS Colitis

**Figure 6:**
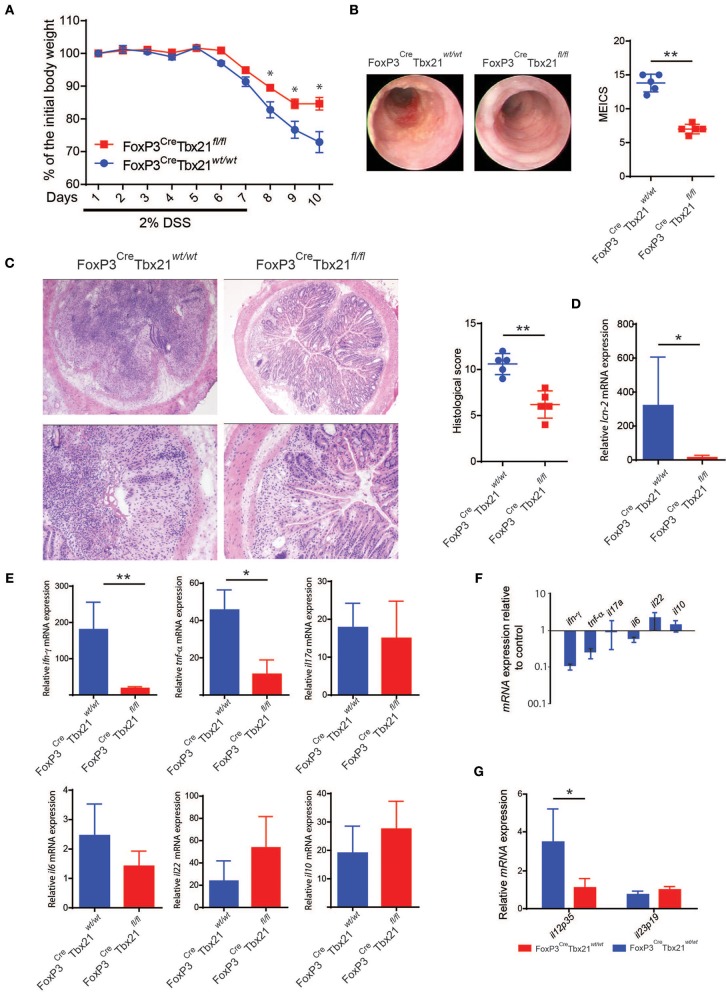
**(A)** Body weight variation relative to the baseline of Treg-specific FoxP3 conditional knockout (FoxP3^Cre^Tbx21^fl/fl^) and control mice (FoxP3^Cre^Tbx21^wt/wt^) upon DSS treatment. Results from one representative experiment out of four performed is shown (mean percentage ±SEM). **(B)** Endoscopic pictures and endoscopic severity grading of knockout and control mice at the end of one representative experiment (Day 10). **(C)** Representative histological section from the colons of FoxP3 knockout and control mice at the end of the experiment and cumulative histologic severity scoring of the experiment shown in **(A)**. *lcn-2* **(D)** and cytokine **(E,G)** mRNA expression in the colon tissue of DSS-treated FoxP3 knockout and control mice. Vertical bars indicate the average ±SD analyzed in the pool of mice from four independent experiments. **(F)** Fold change of cytokine mRNA expression, as indicated, in FoxP3 knockout mice relative to control mice. *p < 0.05; **p < 0.01.

To assess the role of Th1-like Tregs n the development of colitis, we crossed FoxP3^eGFP−Cre^Rosa26^tdRFP^ mice with mice carrying floxed *TBX21* alleles (TBX21^fl/fl^) to obtain a Treg-specific Tbet conditional knockout mouse (from here on indicated as FoxP3^Cre^TBX21^fl/fl^). FoxP3^Cre^TBX21^fl/fl^ and the FoxP3^Cre^TBX21^wt/wt^ mice, used as control, were treated with DSS as described above. FoxP3^Cre^TBX21^fl/fl^ mice showed milder weight loss and lower endoscopic grade of colitis severity as compared to FoxP3^Cre^TBX21^wt/wt^ mice (Figures 6A,B). Accordingly, at the end of the experiment, FoxP3^Cre^TBX21^fl/fl^ mice were characterized by milder colitis than controls, as shown by the histology score (Figure 6C) and the lower expression of the neutrophil-related marker *lcn-2* mRNA (Figure 6D). The cytokine profile analysis showed reduced *ifn*γ and *tnf*α mRNA expression while no significant differences were observed in *il17a, il6, il22*, and *il10* expression (Figure 6E). *ifn*γ and *tnf*α, were the most downregulated cytokines in FoxP3^Cre^TBX21^fl/fl^ mice, with a reduction by 10 and 5-fold respectively (Figure 6F). Finally, *il12p35* but not *il23p19* were reduced in knockout mice as compared to control mice (Figure 6G). These data indicate that the reduced inflammation observed in FoxP3^Cre^Tbx21^fl/fl^ mice was caused neither by a defect in the Th17-mediated immune response nor to an increased expression of anti-inflammatory cytokines but rather by a reduced expression of Th1-related cytokines. Since the frequency of Tregs, ex-Tregs and ConvT, based on the expression of the endogenous fluorescence eGFP and tdRFP, did not differ between Tbet knockout mice and the controls (Supplementary Figure 3A), to investigate whether the milder inflammatory phenotype shown by FoxP3^Cre^Tbx21^fl/fl^ was due to the increased suppressive capacity of Tregs in the absence of Tbet, Tregs from FoxP3^Cre^Tbx21^fl/fl^ and FoxP3^Cre^Tbx21^wt/wt^ were co-cultured with CellTrace®-violet-labeled wild type CD45RB^high^ naïve T cells used as responder cells. In these experiments no difference in the suppressive capacity of wild type and Tbet knockout Tregs cells was observed at each responder-to-suppressor cell ratios (Supplementary Figure 3B). No difference was also observed when Tregs from FoxP3^Cre^Tbx21^fl/fl^ and FoxP3^Cre^Tbx21^wt/wt^ were used *in vivo* in the adoptive transfer model of colitis (Supplementary Figure 3C) as previously reported (24, 25). Finally, the frequency of IL10-expressing Tregs isolated from the colon of either DSS-treated FoxP3^Cre^Tbx21^fl/fl^ or FoxP3^Cre^Tbx21^wt/wt^ did not differ (Supplementary Figures 3D,E). These data suggest that the lower inflammation observed in FoxP3^Cre^Tbx21^fl/fl^ mice is unlikely due to an enhanced suppressive capacity of Tbet-deficient Tregs.

### Conventional Th1 Cells Development Requires the Presence of Functional Th1-Like Tregs

**Figure 7:**
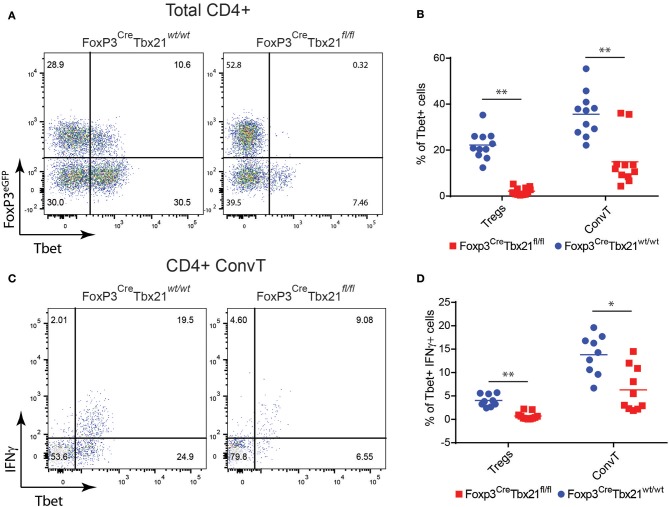
**(A)** Representative dot plots showing FoxP3^eGFP^ and Tbet expression in LP CD3+CD4+ cells isolated from the colons of Treg-specific FoxP3 conditional knockout (FoxP3^Cre^Tbx21^fl/fl^) and control mice (FoxP3^Cre^Tbx21^wt/wt^). **(B)** Frequency of Tbet+ cells among LP Treg and ConvT cells from Tbet knock out and control mice. **(C)** Representative dot plots showing IFNγ and Tbet expression in LP ConvT cells isolated from Tbet knockout and control mice. **(D)** Frequency of Tbet+IFNγ cells among LP Treg and ConvT cells from Tbet knockout and control mice. Numbers in the dot plot quadrants represent the cell subpopulation relative frequency. Horizontal bars in **(B,D)** indicate the mean value and symbols represent each animal analyzed from three independent experiments. *p < 0.05; **p < 0.01.

**Figure 8:**
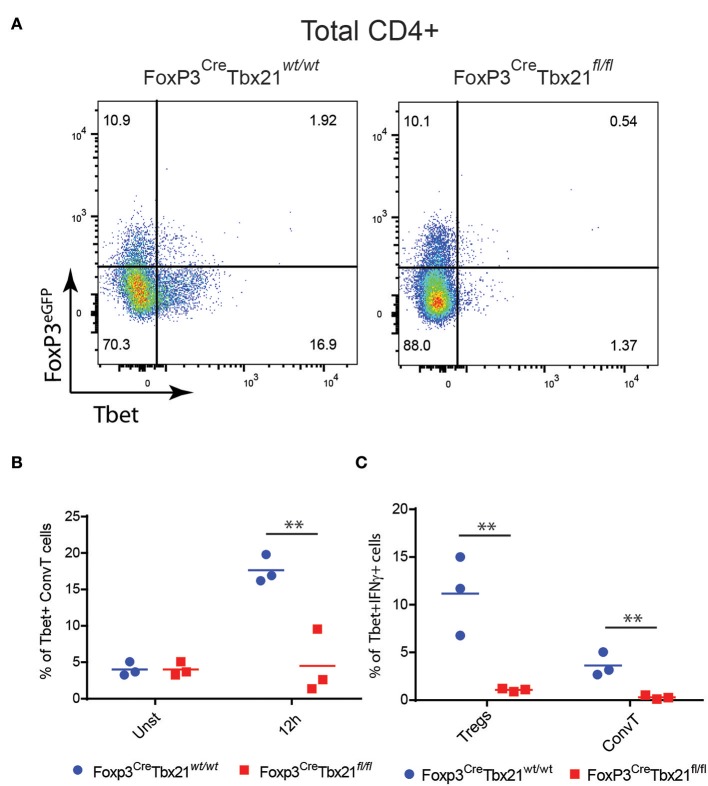
**(A)** Representative dot plots showing the expression of FoxP3^eGFP^ and Tbet in CD3+CD4+ T cells from the spleen of Treg-specific Tbet conditional knockout (Foxp3^Cre^Tbx21^fl/fl^) or control mice (Foxp3^Cre^Tbx21^wt/wt^) polyclonally activated *in vitro* for 12 h. **(B)** Frequency of Tbet+ cells gated on ConvT cells of CD3+CD4+ T cells from Tbet knockout or control mice polyclonally activated *in vitro* for 12 h or left unstimulated. **(C)** Frequency of Tbet+IFNγ+ cells gated on Treg and ConvT cells of CD3+CD4+ Tcells knockout and control mice as in **(B)**. Numbers in the dot plot quadrants represent the relative frequency of cell subpopulations. Horizontal bars in **(B,C)** indicate the mean value of results obtained from three independent experiments. **p < 0.01.

In order to assess whether the phenotype shown by FoxP3^Cre^TBX21^fl/fl^ mice was caused by impaired Th1 cells in the absence of IFNγ-secreting Th1-like Tregs, LPMC from FoxP3^Cre^TBX21^fl/fl^ and FoxP3^Cre^TBX21^wt/wt^ mice were isolated at the end of the DSS treatment and analyzed by flow cytometry. As expected, Tbet-expressing FoxP3+ cells were virtually absent in FoxP3^Cre^TBX21^fl/fl^ mice while about 20% of FoxP3+ cells from FoxP3^Cre^TBX21^wt/wt^ control mice co-expressed Tbet (Figures 7A,B). Interestingly, in TBX21 deficient mice, Tbet+ cells were also reduced by 3-fold among ConvT cells thus indicating that the expression of Tbet in Tregs is required for the development of Th1 cells in the inflamed colon. The expression of IFNγ in ConvT cells was also significantly reduced in the absence of Tbet-expressing Tregs (Figures 7C,D). The reduced expression of Tbet and IFNγ resulted to be specific for CD4+ ConvT cells since their expression remained unaffected in CD8+ cells from FoxP3^Cre^TBX21^fl/fl^ inflamed mice (Supplementary Figures 4A,B). Since LPMC from DSS-treated FoxP3^Cre^TBX21^fl/fl^ mice showed reduced expression of p35IL12, we wondered whether the defective induction of Tbet in ConvT cells in the presence of Tbet deficient Tregs observed occurred independently of innate immune cells. To this end, total CD4+ T cells isolated from the spleen of FoxP3^Cre^TBX21^fl/fl^ or FoxP3^Cre^TBX21^wt/wt^ control mice were polyclonally activated in the absence of antigen presenting cells. After 12 h, the induction of Tbet in ConvT cells from FoxP3^Cre^TBX21^fl/fl^ mice resulted suppressed as compared to controls (Figures 8A,B). In keeping with Tbet expression, IFNγ+ cells were significantly reduced in both Tregs and ConvT cells from FoxP3^Cre^TBX21^fl/fl^ mice as compared to FoxP3^Cre^TBX21^wt/wt^ cells (Figure 8C).

## Discussion

Tregs have been for long considered terminally differentiated, phenotypically stable cells characterized by suppressive capacity. Tregs are crucial for tolerance toward self and harmless antigens. However, the observation that inflammation can induce pro-inflammatory features in Tregs has challenged this concept. The phenotypic changes that may affect Tregs in different inflammatory conditions, are summarized by the terms “stability” and “plasticity.” As for stability, it is meant the loss of FoxP3 expression and the subsequent acquisition of an effector phenotype (26–28). Although this phenomenon has been observed in animal models of immune mediated diseases, it might be circumscribed to a subset of not fully differentiated Tregs in which room for reprogramming in inflammatory conditions still exists (29). Nevertheless, Tregs have been shown to transiently or permanently express T-helper-master regulator transcription factors while maintaining FoxP3 expression. Though, the functional role of these phenotypical changes remains unclear. The expression of Tbet in Tregs has been shown to be required to efficiently control Th1 cells. Koch et al. demonstrated that the IFNγ released in the inflammatory environment induces Tbet in Tregs and that Tbet is required for the expression of the chemokine receptor CXCR3 to address Tregs at the site of inflammation where Th1 cells needs to be kept in check (13). However, in other conditions, the expression of Tbet in Tregs has been associated with the acquisition of a Th1-like pro-inflammatory phenotype characterized by the expression of IFNγ driven by IL12 signaling (14, 15).

The presence of Th1-like Tregs has been observed in different pathologic conditions such as multiple sclerosis (MS) (18), type 1 diabetes (T1DM) (17), autoimmune hepatitis after liver transplant (30), and in animal models of intestinal inflammation (19, 20). Although Tregs have been shown to accumulate in patients affected by IBD, a direct characterization of Th1-like Tregs in this pathologic condition and their functional role in intestinal inflammation is currently missing.

In this study we showed that active IBD is characterized by the accumulation of a sizable fraction of Tregs expressing both Tbet and IFNγ. Since Th1-like Tregs were upregulated in inflamed areas but not in patients with endoscopic disease remission or in uninflamed controls, we hypothesized that Th1-like Tregs could contribute to the inflammatory flares characterizing the relapsing-remitting inflammation observed in IBD. To address this issue, we investigated the role of Th1-like Tregs in the dextrane sodium sulfate (DSS) model of colitis. Similarly to human IBD, Th1-like Tregs accumulated in the inflamed colon. In this model, the upregulation of Tbet and IFNγ in Tregs preceded that of conventional CD4+ T cells (ConvT) and at day 10 the number of Th1-like Tregs represented one third of the total IFN-γ expressing CD4+ T cells. These findings are in agreement with the data reported by Feng et al. in the CBir-1 model of intestinal inflammation (19). In this model the transfer of colitogenic naïve CD4+ T cells specific for the immunodominant commensal antigen, CBir1 flagellin (CBir-1Tg) but not of naïve CD4+ T cells from OTII mice which do not induce colitis in immune-deficient TCRb/d^−/−^ mice was associated to the generation of IFNγ-expressing Th1-like Tregs. Similarly, intestinal inflammation developing in TLR4- and IL10-deficient mice was accompanied by the accumulation of Th1-like Tregs. Thus, the induction of Th1-like Tregs characterizes the development of intestinal inflammation and it can be reproduced in different mouse models (20).

In agreement with previously published data, IFNγ was the main inducer of Tbet in Tregs both *in vitro* and *in vivo* (13–15). Indeed, the IFNγ neutralization in cell cultures of activated CD4+ T cells and the analysis of IFNγ deficient mice treated with DSS, demonstrated that IFNγ is required for the induction of Tbet in the lamina propria Tregs. In contrast to Tbet expression, IFNγ secretion by Tregs has been shown to be dependent on IL12 stimulation and intracellular STAT4 activation. Indeed, the prolonged expression of Tbet in Tregs induces the activation of the IL12 receptor locus in Tregs making these cells responsive to IL12 and inducing the expression of IFN-γ (15). In our *in vitro* system Tregs expressed IFNγ in the absence of IL12, thus indicating that Tbet expression is sufficient to induce the expression of IFNγ, not excluding the possible boosting effect of IL-12 in these cells. Functionally, Tbet expression in Tregs sustained intestinal inflammation since Treg-specific Tbet conditional knockout mice showed milder colitis after DSS treatment as compared to control mice. Tbet knockout mice were characterized by reduced expression of Th1- but not Th17-related cytokines. IL10, which has been shown to downregulate IFNγ expression and to protect from colitis was not increased in Tbet knockout mice (31). However, IL10 expression was not altered in the Tbet knockout Tregs as compared to the wild type controls. The phenotype of Tbet knockout mice was also unlikely due to increased Tregs suppressive capacity since Tbet-deficient Tregs were as suppressive as Tbet proficient Tregs both *in vitro* and *in vivo* in the adoptive transfer model of colitis. However, our data do not rule out the possibility that Tbet-expressing Tregs isolated from the inflamed gut might permanently or transiently lose their suppressive capacity further sustaining the development of inflammation. Finally, the reduced expression of IFNγ among lamina propria CD4+ T cells in DSS-treated knockout mice was not restricted to Tbet-deficient Tregs but it also involved conventional T cells, thus indicating that the Th1 immune response depends on the presence of Th1-like Tregs.

We exclude the possibility that the low Tbet expression in ConvT cells results from a transient activation of the Foxp3 locus during the differentiation of Th1 cells leading to the deletion of Tbet in these cells. Indeed, by using FoxP3 fate mapping reporter mice we were able to monitor the presence of T cells which have transiently expressed FoxP3. In these mice the so called “ex FoxP3+ cells,” marked by the permanent expression of the tdRFP fluorescence even in the absence of FoxP3 expression, represented a negligible fraction of CD4+ T cells and they were excluded from the analysis. Moreover, the frequency of ex-Tregs in Tbet knockout mice did not differ from controls thus indicating that Tbet expression in Tregs does not influence Treg phenotypic stability.

One possible interpretation of these results is that in the very initial phase of inflammation Th1-like Tregs operate as enhancer of the Th1 differentiation process. Different observations sustain this hypothesis. First of all, the relative increase of Tbet-expressing cells was higher among Tregs as compared to ConvT cells *in vitro* and *in vivo* after DSS treatment. Moreover, the induction of Tbet in Tregs preceded that of ConvT cells during DSS treatment when histologic signs of inflammation were barely detectable. Since the expression of Tbet by Tregs was dependent on IFNγ, the ready induction of Tbet in Tregs as compared to ConvT cells might be explained by the FoxP3-dependent upregulation of IFNγ receptor in Tregs vs. ConvT cells (32). Finally, the reduced expression of IFNγ observed in the lamina propria of conditional knockout mice was not limited to the absence of IFNγ-secreting Th1-like Tregs but it was associated to a more general impairment of the Th1 immune response.

The role of Th1-like Tregs as enhancer of Th1 immune response in the early phase of inflammation is also supported by the observation that miR146-deficient Tregs, which are characterized by the upregulation of STAT1-mediated intracellular signaling and IFNγ expression, increased the expression of Th1 cytokines in CD4+ T effector cells in a bone marrow chimera system (32). Moreover, the expression of Tbet and IFNγ in Tregs contributed to the development of Th1-mediated lethal disease in a model of *T. gondii* infection (16).

Interestingly, and in agreement with our data, the upregulation of Tbet and IFNγ in Tregs induced by either mir146 deficiency or *T.Gondii* infection did not affect Treg suppressive activity thus confirming that the Th1 enhancer activity exerted by Tregs during inflammation is not associated with an impaired suppressive capacity.

The next fundamental question is how Th1-like cells enhance the Th1 immune response. It is possible that IFNγ expressed by Tregs directly contributes to the differentiation of conventional Th1 cells in a paracrine manner. However, IFNγ could also promote the differentiation of Th1 cells by upregulating the expression of IL12 in dendritic cells. Accordingly, IL12p35 but not IL23p19 was downregulated in the lamina propria of Tbet conditional knockout mice.

In conclusion, based on our data, we propose a model in which during the development of intestinal inflammatory flares, Th1-like Tregs enhances the initial phase of inflammation by promoting the development of Th1 cells. In this this context, Th1-like Tregs would represent an another source of IFNγ-producing cells involved in colitis development. Indeed, in addition to the classic Th1 differentiation from a naïve T cells, Th1 cells have been shown to derive from Th17 cells conversion in different inflammatory conditions including colitis. Therefore, the generation of Th1-like Tregs would represent just another moment of a general Th1-skewing process involving different T cell sub-types during the intestinal inflammation (33, 34).

At the same time, Tregs maintain their suppressive activity on effector cell proliferation thus avoiding an excessive inflammatory response. A dysregulation of this process, causing a lower threshold of activation of the Treg-mediated pro-inflammatory activity, might contribute to the generation of chronic inflammation in the gut and to the pathogenesis of IBD.

## Data Availability

The datasets generated for this study are available on request to the corresponding author.

## Ethics Statement

The studies involving human participants were reviewed and approved by Tor Vergata University Hospital, Rome. Protocol number: 154/12. The patients/participants provided their written informed consent to participate in this study. The animal study was reviewed and approved by Authorization No: 324/2006-PR issued by the Ministry of Health on 29/03/2016.

## Author Contributions

MD and AR contributed to the experimental design and performed the experiments. EF performed the experiments. FF, CS, and SO analyzed the lamina propria cells from human samples. FC collected the bioptic samples from IBD patients and reviewed the manuscript. AF analyzed the data. GM contributed to the study design. H-JF provided the Rosa26tdRFP reporter mice. MF designed the experiments, analyzed the data, and wrote the manuscript.

### Conflict of Interest Statement

The authors declare that the research was conducted in the absence of any commercial or financial relationships that could be construed as a potential conflict of interest.
