# Supplementary figures and images for: Tbet Expression in Regulatory T Cells Is Required to Initiate Th1-Mediated Colitis
Source: Front Immunol. 2019 Sep 11;10:2158. doi: 10.3389/fimmu.2019.02158 (PMC6749075; doi:10.3389/fimmu.2019.02158)

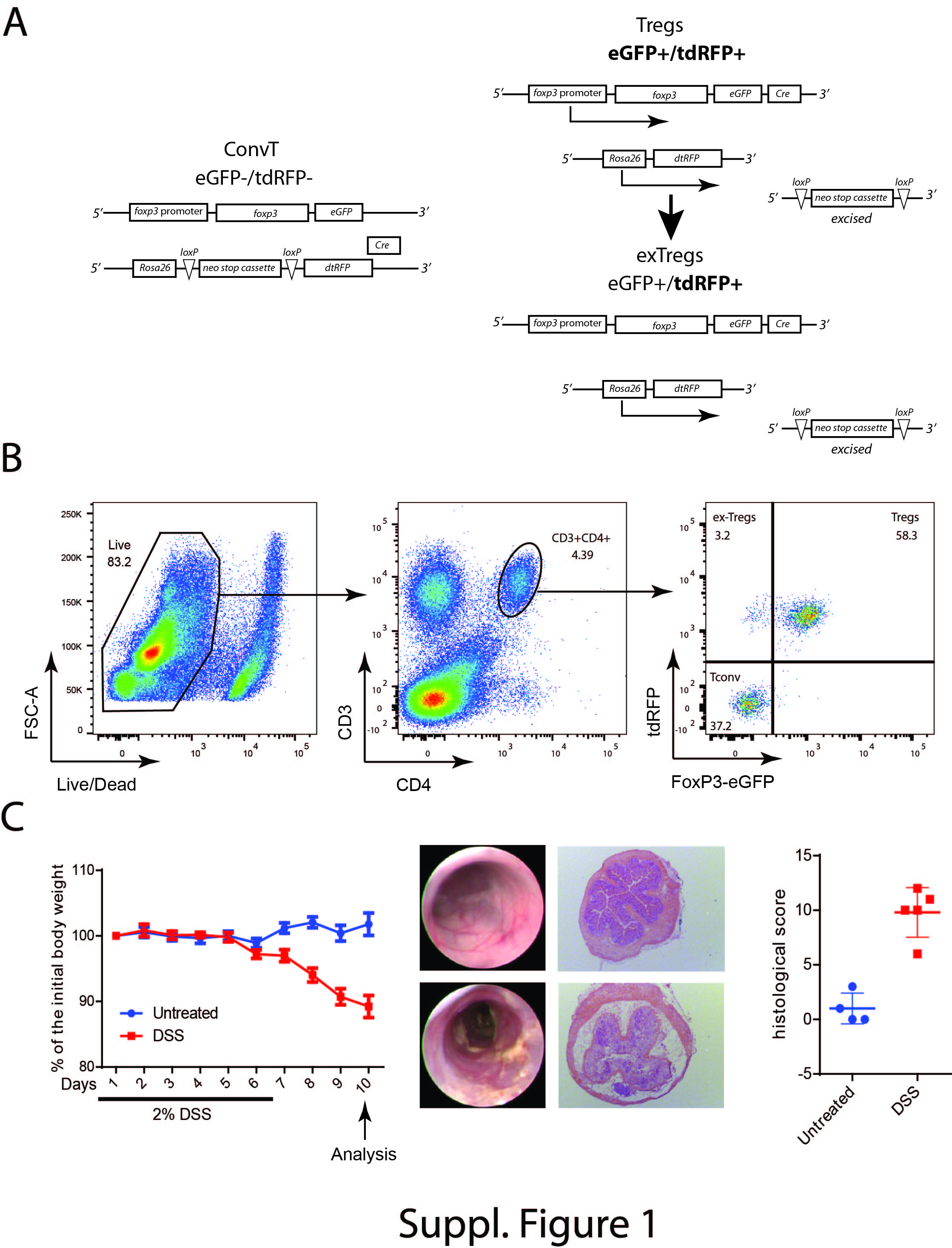

Supplement: Supplementary file 1 [file Image_1.jpg]

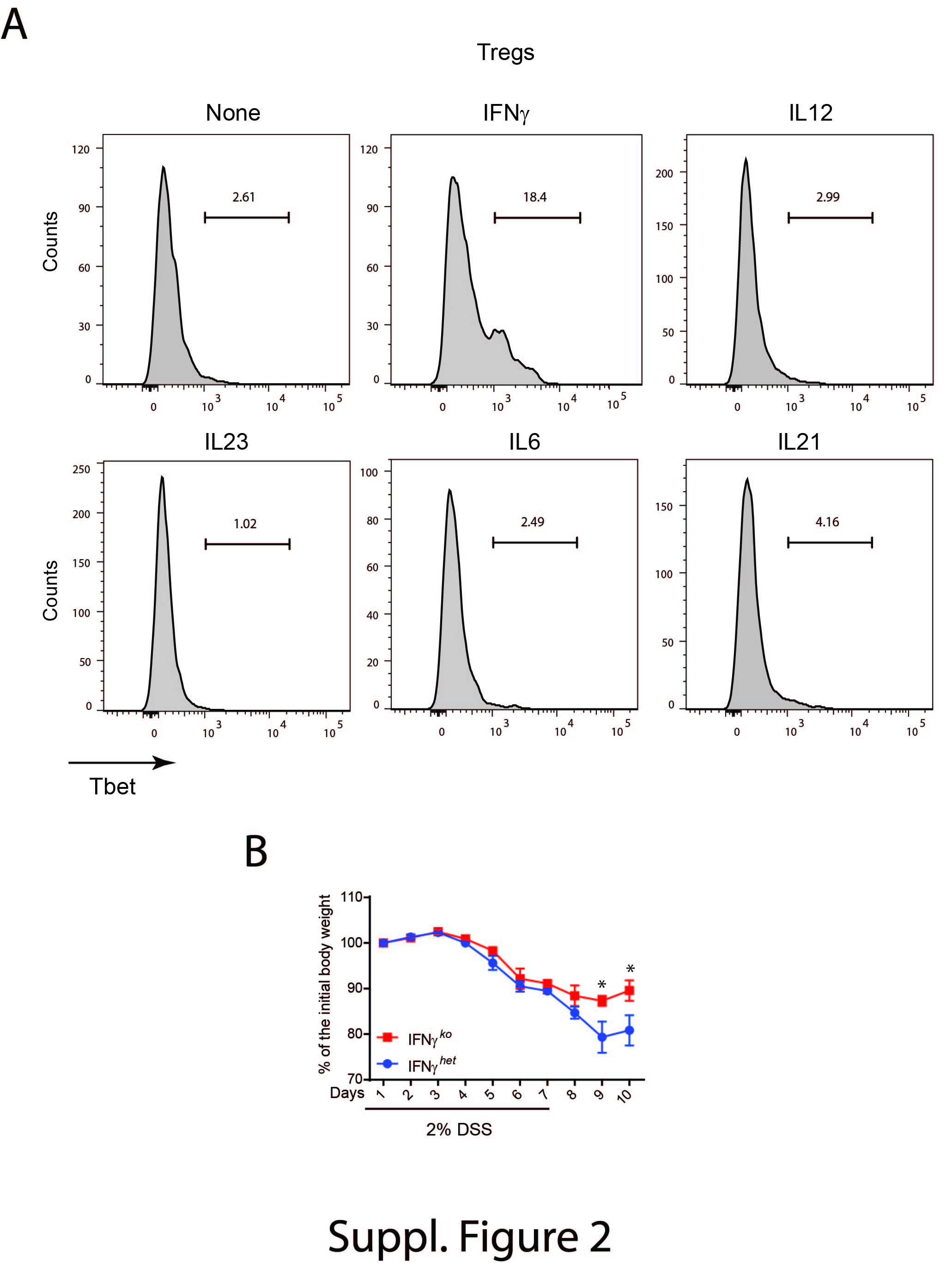

Supplement: Supplementary file 2 [file Image_2.jpg]

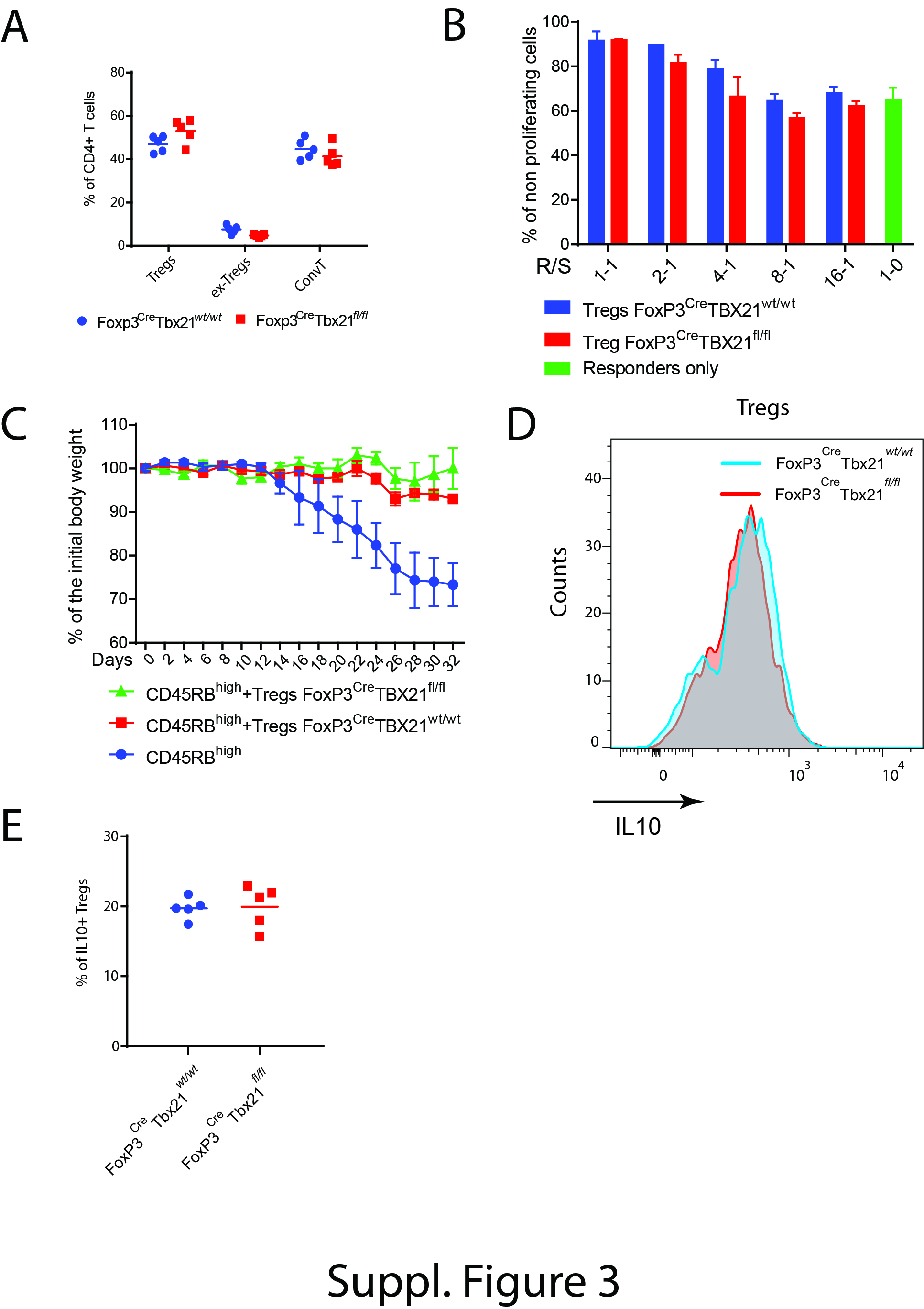

Supplement: Supplementary file 3 [file Image_3.TIF]

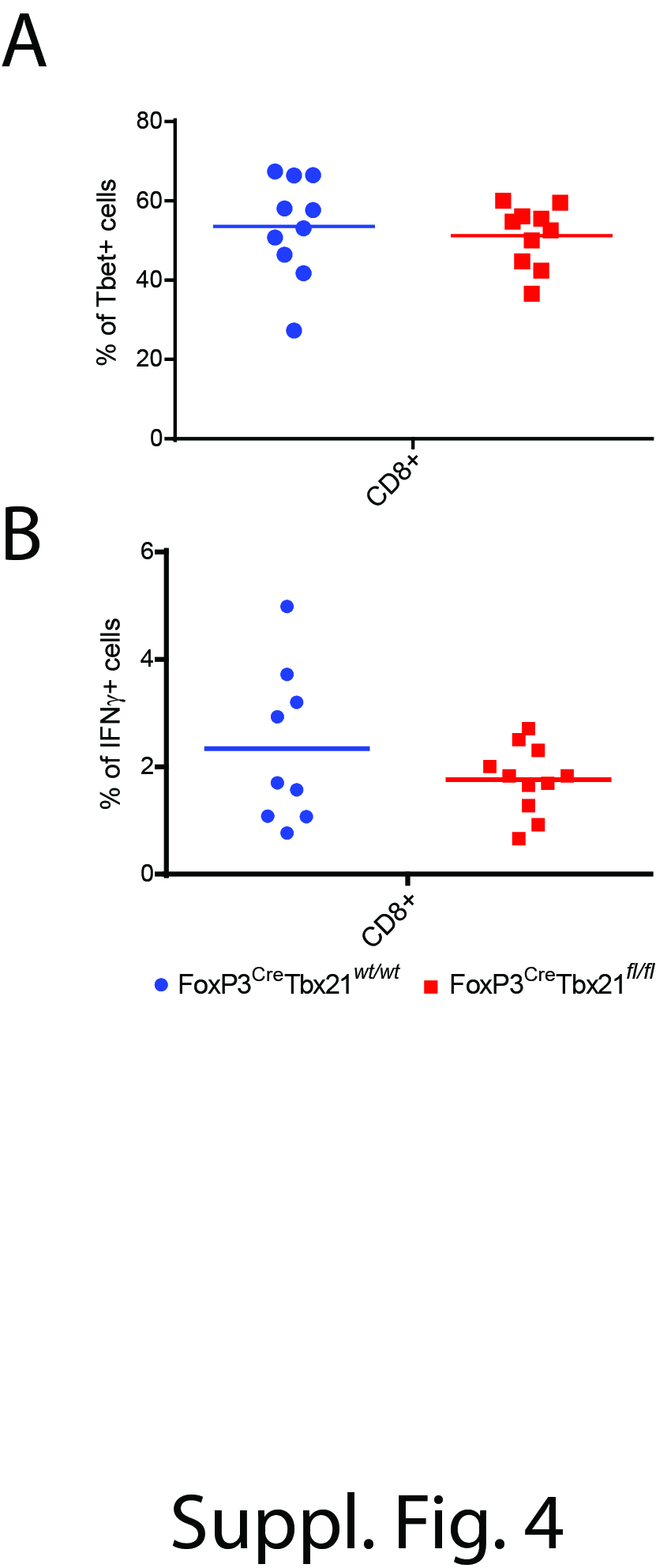

Supplement: Supplementary file 4 [file Image_4.TIF]
