# Supplementary material for: Tbet Expression in Regulatory T Cells Is Required to Initiate Th1-Mediated Colitis
Source: Front Immunol. 2019 Sep 11;10:2158. doi: 10.3389/fimmu.2019.02158 (PMC6749075; doi:10.3389/fimmu.2019.02158)
Supplement: Supplementary file 5 [file Table_1.DOCX]

**Supplementary materials:**

**Supplementary figure 1:** (**A**) Description of *foxp3* and *rosa26* loci as modified in the Foxp3 fate mapping reporter mice. The activation of the Foxp3 promoter causes the co-expression of FoxP3, the enhanced green fluorescent protein (eGFP) and the recombinase Cre. Cre expression leads to the excision of the Neo stop cassette permanently activating the expression of the tandem dimer red fluorescence proteins (tdRFP). Tregs which lose the expression of FoxP3 retain the expression of tdRFP (exTregs). (**B**) Gating strategy adopted for the analysis of regulatory (Tregs) and conventional T cells (ConvT) in the FoxP3 fate mapping reporter mice based on the expression of the endogenous fluorescence. Tregs were defined as eGFP/dtRFP double positive and ConvT cells as eGFP/tdRFP double negative CD3+CD4+ T cells. eGFP-tdRFP+ cells indicate Tregs which have lost FoxP3 expression (i.e. ex-Tregs) (**C**) Induction of colitis by oral administration of dextran sodium sulphate in the FoxP3 reporter mice: mean percentage variation ±SEM of the weight relative to baseline in the DSS-treated and untreated control groups (**C** left panel). Representative endoscopic and histologic pictures of the colon from treated and untreated mice at the end of the experiment, day10 (**C** central panel); histologic score quantification of a representative experiment of three performed. Each square or circle represent one mouse (**C** right panel).

**Supplementary figure 2:** (**A**) Representative histograms showing the expression of Tbet in FACS sorted CD4+FoxP3+ Tregs polyclonally activated in vitro for 24 hours in the presence of IL2 alone or in combination with either IFNγ or IL12 or Il23, or IL6 or IL21 as indicated. Representative results of three independent experiments are reported. Numbers in the histograms epresent the cell subpopulation relative frequency. (**B**) Over the time mean percentage variation of the body weight ±SEM relative to the baseline of IFNγ^ko^ and IFNγ^het^ treated with DSS as indicated. Results from one representative experiment out of four performed is shown.

**Supplementary figure 3:** (**A**) Frequency of Tregs (eGFP+tdRFP+), ex-Tregs (eGFP-tdRFP+) and ConvT (eGFP-tdRFP-) cells in the lamina propria of DSS treated from Treg-specific Tbet knockout (Foxp3^Cre^Tbx21^fl/fl^) or control (Foxp3^Cre^Tbx21^wt/wt^) mice. Horizontal bars indicate the mean value and symbols represent each animal analyzed. (**B**) *In vitro* suppression assay: FACS sorted CD4+FoxP3- responder cells and Tbet-deficient or control Tregs were co-cultured *in vitro* at different responder-to-suppressor (R/S) ratios as indicated. Bars indicate the relative frequency of non-proliferating cells based on CellTrace® dilution after 48 polyclonal activation. Bars indicate mean ±SD. Results from one representative experiment out of three performed are shown. (**C**) Adoptive transfer model of colitis: FACS sorted CD4+CD45RB^high^ cells were injected intraperitoneally in RAG1-deficient mice alone or together with Tregs isolated from Tbet knockout or control mice. Mean percentage variation ± SEM of the body weight relative to the baseline of the three groups of mice over the experimental time frame. (**D**) Representative histograms showing the expression of IL10 in Tregs isolated from the lamina propria of DSS-treated knockout and control mice. (**E**) Frequency of IL10 cells among LP Tregs from Tbet knock out and control mice. Horizontal bars in B and D indicate the mean value and symbols represent each animal analyzed.

**Supplementary figure 4**: Percent of Tbet+ (**A**) and IFNγ+ (**B**) cells in the LP CD8+ cells from Tbet knockout and control mice. Numbers in the dot plot quadrants represent the cell relative frequency. Horizontal bars indicate the mean value and symbols represent each animal analyzed from three independent experiments.

**Supplementary table 1:** Disease characteristics of CD and UC patients analyzed in Figure 1

**Supplementary table 2:** Primer sequence table.

| UC (n=12) |  |
| --- | --- |
| Age; n (range) | 53 (27-73) |
| Active smoker; n (%) | 0 (0) |
| Extent*, n (%) |  |
| E1 | 2 (16.7%) |
| E2 | 4 (33.3%) |
| E3 | 6 (50.0%) |
| ISS; n (%) | 0 (0%) |
| anti-TNFs n (%) | 2 (16.7%) |
| Mayo endoscopic score 0-1; n (%) | 5 (41.7%) |

**Suppl. Table 1**

| CD (n=13) |  |
| --- | --- |
| Age, n (range) | 64 (44-80) |
| Active smoker; n (%) | 1 (15.4) |
| Localization*; n (%) |  |
| L1 | 2 (15.4) |
| L2 | 5 (38.5) |
| L3 | 6 (7.7) |
| Behavior*; n (%) |  |
| B1 | 2 (15.4) |
| B2 | 10 (76.9) |
| B3 | 1 (7.7) |
| ISS; n (%) | 4 (30.8) |
| Anti-TNFs; n (%) | 2 (15.4) |

*accordingly to Montreal classification

**Suppl. Table 2**

| Gene | Sense | Anti-sense |
| --- | --- | --- |
| *β-actin* | 5′-AAG ATG CCC AGA TCA TGT TTG AGA CC-3′ | 5′-AGC CAG GTC CAGA CGG AT-3′ |
| *Tnfα* | 5′-ACC CTC ACA CTC AGA TCA TC-3′ | 5′-GAG TAG ACA AGG TAC AAC CC-3’ |
| *Ifnγ* | 5′-CAA TAG ACG CTA CAC ACT GC-3′ | 5′-CCA CAT CTA TGC CAC TTG AG-3′ |
| *il17a* | 5′-TCA GAC TAC CTC AAC CGT TC-3′ | 5′-TTC AGG ACC AGG ATC TCT TG-3′ |
| *il6* | 5′- CCA TAG CTA CCT GGA GTA CAT G -3′ | 5′- TGG AAA TTG GGG TAG GAA GGA C-3′. |
| *il22* | 5′- TGA GGT GTC CAA CTT CCA GCA GC -3′ | 5′- GTT TCT CCC CGA TGA GCC GG-3′ |
| *Il10* | 5´-TAC CTG GTA GAA GTG ATG CC-3´ | 5´-TAG ACA CCT TGG TCT TGG AG-3´ |
